# Validating nasogastric tube placement with pH testing: A randomized controlled trial protocol

**DOI:** 10.1016/j.conctc.2024.101312

**Published:** 2024-05-22

**Authors:** Stefano Mancin, Pietro Stallone, Valeria Siro, Manuela Pastore, Daniela Cattani, Diego Lopane, Alessandra Dacomi, Francesco Carlo Tartaglia, Alessandro Bellone, Francesca Serazzi, Georges Laffoucriere, Chiara Coldani, Giuseppina Tomaiuolo, Beatrice Mazzoleni

**Affiliations:** a Department of Biomedicine and Prevention, University of Rome “Tor Vergata”, Rome, Italy; b Department of Biomedical Sciences, Humanitas University, Pieve Emanuele, Milan, Italy; c IRCCS Humanitas Research Hospital, Rozzano, Milan, Italy

**Keywords:** Nasogastric tube, pH testing, Randomized controlled trial, Innovative technologies, Enteral nutrition

## Abstract

**Background:**

Enteral nutrition (EN) is preferred when oral feeding is not possible. The use of the Nasogastric Tube (NGT) ensures rapid and low-risk nutrient administration. However, confirming the placement through chest radiography, besides delaying the initiation of nutritional therapy, exposes patients to radiation. The pH test of gastric aspirate provides a quicker check for NGT placement, but its reliability is compromised by challenges related to aspirating gastric secretions.

**Study objective:**

The main objective of this study is to assess the high-performance placement of NGTs for nutritional purposes, optimizing the evaluation of correct insertion through pH testing using an electronic pH meter. Additionally, the study aims to evaluate patient tolerance to the intervention.

**Materials and methods:**

This single-center RCT will include 150 EN candidate patients divided into three groups. Each group will use distinct NGTs, evaluating placement through pH testing and chest radiography for safety. Tolerance, complications related to NGT placement, and costs will be assessed, with data collected anonymously through a secure electronic database.

**Ethical considerations:**

authorization no. 3624, Territorial Ethical Committee Lombardy 5, October 20, 2023.

**Implications and perspectives:**

This protocol introduces innovative technologies, such as advanced NGTs and an electronic pH meter, aiming to optimize enteral nutrition management. This RCT focuses on replacing X-rays as the primary method for verifying NGT placement, thereby reducing costs, time, and patient exposure to radiation. Data analysis may provide insights into managing patients on pH-altering medication. Implementing innovative technologies has the potential to reduce errors and improve economic efficiency and process sustainability.

## 1 Introduction

Hospital malnutrition is a condition characterized by an insufficient intake of nutrients, leading to a compromise in health status and quality of life [1]. It is estimated that in Europe, the prevalence of malnutrition related to acute or chronic diseases in hospitalized patients is around 30 % [1]. Moreover, malnutrition can lead to negative clinical outcomes [2] such as: immune system issues [3], difficulty in wound healing [4], loss of muscle mass [5], longer hospital stays [6], higher hospitalization costs [7], and increased mortality [8]. To prevent and treat hospital malnutrition, it is crucial to assess the risk of nutritional deficiency or the presence of malnutrition during all stages of hospitalization, using validated and standardized screening tools [9]. This not only allows for planning focused on prevention but also early treatment of a potential malnourished state [10]. Additionally, nutrition and hydration are considered primary needs of the patients, directly involving nursing staff in nutritional care at various levels [11]. In light of this, when physiological oral feeding is not possible, enteral nutrition should always be considered as the elective nutritional treatment, not only for the maintenance of gastrointestinal tract homeostasis but also due to the lower risk of complications [12] and reduced costs [13]. Enteral nutrition involves the introduction of food into the digestive system through a nasogastric tube, nasojejunal tube, surgical gastrostomy, or percutaneous endoscopic gastrostomy (PEG) [14,15]. It has shown better clinical outcomes when started early [16], promoting better wound healing [17], improved modulation of the inflammatory response [18], prevention of intestinal atrophy [19], reduction of complications, and decreased mortality [20,21]. The first-line nutritional support commonly used for nutritional purposes is NGT [22]. This medical device, whose placement and management is the responsibility of the nursing staff, is a safe medical device and allows the initiation of enteral nutrition administration in a reduced timeframe [23], depending on the waiting times for a control chest x-ray (current Gold Standard) of individual hospitals to verify its correct placement. Recent studies [24,25] suggest that the evaluation of gastric aspirate using the pH test could be a valid alternative to confirm the placement of the NGT (pH ≤ 5.5). These studies also indicate the opportunity to use digital equipment [26] for such measurement, overcoming the limitations of traditional reactive strips. Indeed, the interpretation of pH using reactive strips could be imprecise [27,28], and this could lead to possible interpretation errors by healthcare professionals. Several studies have shown that the interpretation of pH with the most common reagent strips involves possible biases in distinguishing between pH values of 5.0 and 6.0. Furthermore, pharmacological treatments with antacids and the intake of food could increase gastric pH, leading to possible biases in the obtained result [29,30]. However, considering the indications from a recent study [31], it is necessary to evaluate possible difficulties related to the procedure of aspirating gastric secretions for the pH test. Indeed, as expressed by several authors [32], the use of standard NGTs with a small calibre (8–12 CH) and without a second lumen dedicated to aspiration [32] sometimes involves difficulties in aspirating gastric secretions, increasing the risk of error; in the face of these problems, the use of chest radiography is indicated to confirm correct placement [33,34]. However, chest radiography involves exposure to radiation [[33], [34], [35]], an additional cost, and a delay in the start of feeding or any pharmacological therapy [36]. These delays contrast with the primary objective of feeding through the NGT, which is to improve the nutritional intake of patients at high risk of malnutrition quickly [37].

In the current context, the verification of the correct placement of the nasogastric tube is carried out through a rigorous multidisciplinary protocol. The procedure involves the aspiration of at least 5 ml of gastric secretion and the subsequent evaluation by pH test. In case of the procedure being unsuccessful, the placement is defined through chest X-ray. Based on the collected data, the percentage of patients in whom it was possible to successfully apply this protocol was 42% of all placements performed. This result highlights a limited success rate, largely associated with intrinsic difficulties encountered during the aspiration phase. These difficulties are frequently linked to the current use of nasogastric tubes, demonstrating significant complexity in implementing the multidisciplinary protocol. The data aligns with current scientific literature, indicating potential limitations in the application of this procedure. This could not only compromise nutritional benefits for the patient but also hinder cost optimization from an economic sustainability perspective. To date, existing scientific literature lacks clinical studies addressing the highlighted issues, both in terms of overcoming difficulties in gastric aspiration and adopting the use of electronic pH meters [[38], [39], [40], [41], [42], [43], [44]].

### 1.1 Study Objectives

The aim of this study is to evaluate the placement of NGT for nutritional purposes according to the best standards of safety and quality by developing a multidisciplinary protocol for this procedure. Through the use of NGTs that can guarantee greater aspiration performances with the same caliber, it would be possible to have a better aspiration of the gastric aspirate (≥5 ml) which would guarantee the possibility of carrying out the pH test in order to ensure correct positioning of the NGT.

### 1.2 Primary endpoint

Evaluate the correct positioning of the NGT for enteral nutrition assuming an aspiration of the gastric aspirate of at least 5 ml in order to be able to carry out the pH test using an electronic pH meter (measurement carried out at the first positioning of the NGT).

Ability of the pH test to identify incorrect positioning of the SNG as well as the result of the chest x-ray. Furthermore, in the event of a doubtful RX result or one that does not identify the correct positioning, the pH test is able to detect the incorrect positioning.

### 1.3 Secondary endpoint

The first secondary outcome to evaluate is the sensitivity/specificity of the pH test compared to the current Gold Standard (chest X-ray), also in consideration of clinical variables such as: food intake (time calculated in minutes from the last food intake, guaranteeing a minimum time to perform the test of at least 1 h) and taking drugs that affect gastric pH (proton pump inhibitors and antacids). Secondly, to evaluate the tolerance of the different NGT models used through pain monitoring (evaluated through the NRS scale at 24-48-72 h after placement and where an NRS score greater than ≥3 will be considered as a threshold value to identify poor tolerance). Thirdly, to evaluate the starting time of nutritional therapy from the request for placement of the NGT by the attending physician (calculated in minutes). Lastly, to evaluate possible complications associated with the positioning of the NGT and arising during the hospitalization period, such as: 1) mispositioning of the NGT (assessed with Chest X-ray); 2) tracheobronchial aspiration; 3) aspiration pneumonia 4) lesions of the oesophageal mucosa and of the upper airways (for points 2,3,4 evaluations by clinical examination, blood tests and instrumental tests requested by the attending physician).

### 1.4 Other outcomes of interest

Evaluation of cost reduction compared to the current gold standard (chest radiography). Costs related to the entire NGT placement procedure (cost of NGT, personnel, clinical procedure for monitoring correct placement) will be evaluated. This calculation will be expressed in Euros.

### 1.5 Safety outcomes

At each placement of NGT, a check will be carried out by means of a pH test with an electronic pH meter associated with a chest x-ray in order to evaluate the correct or not correct positioning of the device.

## 2 Methods

### 2.1 Study design

This study is a randomized clinical trial regarding the efficacy of different NGTs in aspiring gastric secretions so as to assess correct NGT placement by pH monitoring and reduce the use of chest X-ray (CXR) in everyday clinical practice.

### 2.2 Study procedures

**Fig. 1:**
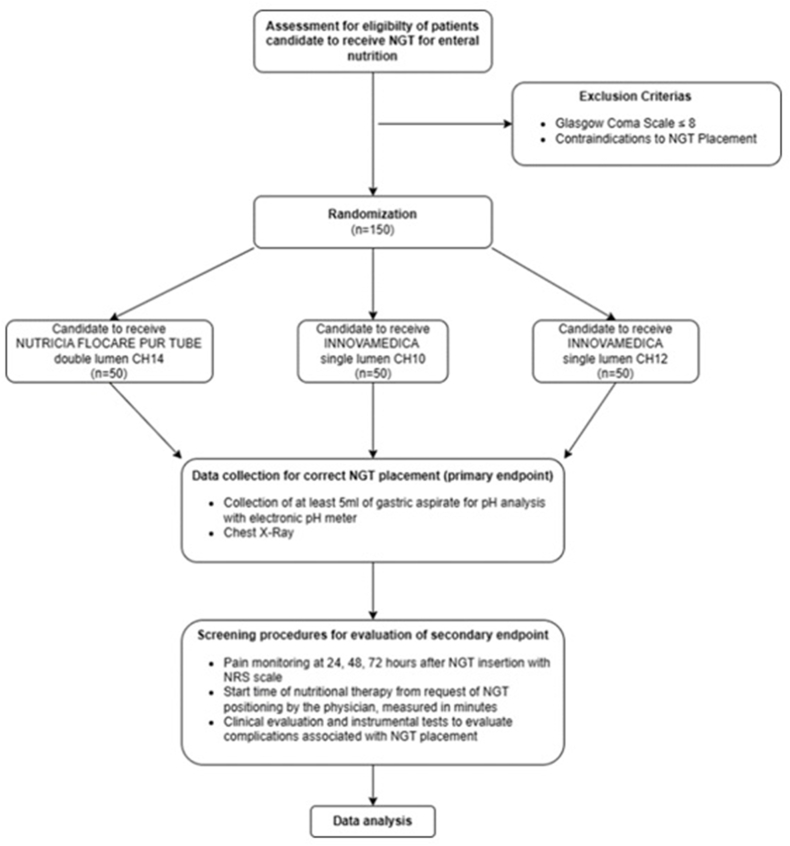
Sample enrollment and study Procedures **Legend.** NGT= nasogastric tube; CH=Charrier; NRS= numerical rating scale.

Participants will be enrolled in three study arms, assigned according to the defined randomization criterion, to evaluate three different NGT models along with the use of an electronic pH meter. The first NGT is the *NUTRICIA FLOCARE PUR TUBE CH14 double lumen*, which features an additional lumen to the nutritional lumen specifically for pH aspiration. The other two NGTs are the *INNOVAMEDICA single lumen CH10 AVANOS_ST CH10* and the *INNOVAMEDICA single lumen CH12 AVANOS_ST CH12*, which have a single lumen. In all three study arms, the determination of the correct positioning (primary outcome) will take place through the monitoring of the gastric pH (gastric aspirate 5 ml; pH value ≤ 5.5)^46^ through the use of an electronic pH meter (pH Strips & Reader Innovamedica model pH X-act) which will be compared with the CXR (current gold standard). The comparison with the CXR will allow to evaluate the sensitivity and specificity of the pH test. An important aspect of the study is the evaluation of the time efficiency in starting nutritional therapy. This is measured in minutes and aims to determine if the pH test can reduce the time taken for NGT positioning evaluation compared to the current 1440 min required for chest X-ray assessments. The time frame is obtained from the evaluation of the service at IRCSS Humanitas Research Hospital. Furthermore, Patient tolerance to the NGT will be assessed at 24, 48 72 h post-placement using the NRS scale. This will help identify pain levels and characteristics, to evaluate whether different NGT calibers (CH) affect patient tolerance. Throughout the entire period of hospitalization of the patient, complications associated with the positioning of the NGT will be recorded. These include mispositioning of the NGT (assessed by chest x-ray); tracheobronchial aspiration; aspiration pneumonia; lesions of the oesophageal mucosa and upper airways (assessed by clinical examination and instrumental tests requested by the attending physicians). During the post-clinical phase, an economic analysis will be conducted to evaluate potential cost savings from using the pH test for correct NGT positioning, with respect to X-ray use. This includes analysing costs of auxiliary personnel (based on transport times to radiology, calculated as Euro/hour), professional fees for performing chest Xrays, and the cost of the Xrays themselves. A graphical representation of the study procedures is depicted in Fig. 1.

#### 2.2.1 Recruitment of study participant

Over a time period of at least 6 months, patients admitted to the wards of IRCCS Humanitas Research Hospital will be subsequently enrolled in three study groups according to the inclusion/exclusion and randomization criteria described further on.

*Inclusion Criteria*: Eligibility criteria for a study involving NGT placement for enteral nutrition in hospitalized patients are as follows: Firstly, patients considered for NGT insertion should not have any current complications that may interfere with the placement of the tube, particularly those related to anatomical challenges or known risks associated with elective NGT placement. Secondly, only legally adult patients are eligible to participate. Finally, it is imperative that all participants consent to the use of their personal data for research purposes, which is formalized through the signing of a consent form. These criteria are essential to ensure the selection of suitable candidates for the study, minimizing potential complications and ensuring informed participation.

*Exclusion Criteria*: Patients who, at the time of positioning, present neurological complications such as to involve a significant alteration of the level of consciousness measured by the Glasgow Coma Scale (GLS) with a value ≤ 8 will be excluded. Patients who have contraindications to NGT placement, such as basal skull fractures; Maxillo facial disorders; Unstable cervical spinal injuries; Nasal/pharyngeal/oesophageal obstruction or ulceration; Choanal atresia; Tracheoesophageal fistula; Oesophageal/pharyngeal pouch; Oesophageal stricture or other abnormalities of the oesophagus; Oesophageal tumours or have undergone oesophageal surgery; Oropharyngeal tumours or have undergone oropharyngeal surgery; Post laryngectomy; Actively bleeding oesophageal or gastric varices; Gastric outflow obstruction; Intestinal obstruction; pregnant women.

#### 2.2.2 Assignment to investigation groups

The randomization will be conducted using sealed envelopes prepared by a dedicated statistician for the study. Upon opening each envelope, a dual signature will be applied to ensure that the randomization procedure adheres to statistical requirements. The process will be managed to maintain confidentiality, and none of the professionals involved in the clinical procedures of the study will have access to randomization information. The study will employ a specially designed procedure to ensure the integrity and statistical validity of the randomization process.

#### 2.2.3 Subject withdrawal procedures

The patient has the right to decide to abandon the study at any stage or to withdraw his consent by communicating the decision to the principal investigator or other investigator who will become reversible at each stage of the study.

#### 2.2.4 Methods for minimizing bias

The pH measurement of the gastric aspirate, as indicated by several authors, will be evaluated at least 1 h after food intake. If the patient is taking antacids or proton pump inhibitors (PPI), we hypothesize that the pH data may present possible alterations; in this regard we will record variables of interest, reporting everything in the CRF form, including the intake of antacids and/or PPI drugs, daily dosage, and time since the last intake of the drug at moment of pH measurement. We believe the latter evaluations are necessary as to date there are no clear guidelines, protocols or clinical studies that highlight how to deal with such possible clinical biases. Furthermore, the calibration of the instrument will be carried out daily following the instructions and kit which are provided by the manufacturer by a responsible and trained healthcare professional. Additionally, before the start of the study, a specific 2-h training session will be conducted to train healthcare professionals on the study procedures and the use of the pH meter. This course will be delivered by a specialist nurse, a nutritionist, and by the clinical quality and risk department of the hospital.

#### 2.2.5 Clinical investigation intervention

*Medical device.* This randomized clinical trial will include three study arms to which patients will be assigned according to the enrolment criteria set out in the research protocol. Each arm of the study will involve a different type of NGT. The types of NGTs are reported in section 1.1. These two NGTs have a single lumen and are made of specific materials that give greater performance. In all three study arms, the determination of the correct positioning (primary outcome) will take place through the monitoring of the gastric pH (gastric aspirate 5 ml; pH value ≤ 5.5) through the use of an electronic pH meter (pH Strips & Reader pH X-act) which will be compared with the chest X-ray (current gold standard).

*Labelling and supply.* All electro medical equipment (pH meter) for this study will be provided free of charge for testing purposes by the respective manufacturers/suppliers, while medical devices (NGTs) will be provided free of charge.

*Storage conditions.* The NGT devices can be kept in the common storage environments used for the principals. A total of two pH metres will be assigned to each ward. The management and storage of the device will be carried out by the analysis laboratory of the structure in which the trial is being performed.

#### 2.2.6 Discontinuation or modifications of the intervention

Interruption of treatment foreseen in case of impossibility to carry out the NGT positioning procedure.

#### 2.2.7 Compliance with clinical investigation intervention

Correct positioning will be monitored by pH testing with an electronic pH meter and chest X-ray. Any acute complications related to the positioning of the nutritional aid and the patient's tolerance during the period of hospitalization will also be monitored.

#### 2.2.8 Clinical investigation specific preventive measures

The pH measurement of the gastric aspirate will be evaluated at least 1 h after food intake. If the patient is taking antacids or proton pump inhibitors (PPI), we hypothesize that the clinical data (pH test) may present possible alterations; in this regard we will evaluate as variables of interest, reporting everything in the CRF form, intake of antacids and/or PPI drugs, daily dosage and, when the pH is measured, time since the last intake of the drug. We believe the latter evaluations are necessary, since this data is a possible clinical bias and, in the literature, there are no clinical studies, protocols or guidelines that answer this question.

#### 2.2.9 Concomitant treatments

It is important to consider pharmaceuticals or medicines or supplements or foods that can alter gastric pH prior to the procedure.

### 2.3 Medical device accountability

This study does not include specific procurement or storage of materials (NGTs). With regard to the procurement and storage of electromedical devices to be used (electronic pH meters), a minimum supply of two instruments will be required which will be managed in compliance with the regulations in place at the IRCCS Humanitas Research Hospital Rozzano (MI), the Clinical Engineering service and according to the manufacturer's specifications. Based on the number of electronic pH meters supplied by the manufacturer, hospital departments will be assigned electromedical device (electronic pH meter and reagent strips) in order to carry out the tests.

### 2.4 Return or destruction of the medical device

As regards the NGT in use, if they are removed after positioning during the hospitalization period, the disposal will be the responsibility of the hospital through the procedure that regulates special hospital waste. The pH meters in use, being supplied free of charge by the distributing company, will be returned at the end of this study.

### 2.5 Statistical considerations

#### 2.5.1 Determination of sample size

Under the hypothesis that with standard method only 42 % of catheter positioning of the NGT allow the measurement of the pH of the gastric aspirate, we want to demonstrate, with a power of 80 % and a type I error of 5 % that the 3 studied NGT allow measurement of pH in at least 62 % of positioning. Therefore, the study need 47 patients in each arm. These numbers were gathered from the current data available at IRCCS Humanitas Research Hospital. This sample size also allows us to see a difference in discomfort between the three study groups as the difference in NGT calibre could be tolerated differently (NRS scale). Following this analysis, a total of 150 patients, subdivided into 50 patients per branch, have to be included in the study.

#### 2.5.2 Analysis populations

All randomized patients will be evaluated as Intent-to-Treat (ITT) bases, so all randomized patients classified on the basis of the treatment arm they were randomized to. If any patients, for any reason switch to another group, also a per protocol (PP) analysis wil be performed, and the patients will be considered in the treatment arm they received. Patients with pH measurement will be considered as the measured population (MP).

#### 2.5.3 Statistical analysis

Continuous variables following a symmetric distribution will be presented with mean (SD), while skewed variables will be presented with median (IQR). Ordinal variables will be presented with median (IQR) and categorical variables will be presented as n (%). For primary endpoint, the percentage of catheter positioning allowing pH measurement will also be described with 95 % confidence interval. Differences among the three groups will be explored with chi square test for categorical variables, or one-way ANOVA or Kruskal Wallis test, for continuous variable, depending on their distribution shape. MP patients will also be analysed in order to explore the existence of a different pH threshold to identify the correct catheter positioning in case of use of antiacids-PPIs drugs. All analysis will be performed with Stata version 17 or superior. Statistical significance was set to p < 0.05.

## 3 Implications and Perspectives

According to international guidelines, the correct positioning of a NGT may be checked by testing the pH of aspirate, or by X-ray. The pH test method is preferrable due to lower costs as well as absence of radiation exposure for the patient. Unfortunately, pH testing is not always feasible, often due to difficulties in aspiration of current NGTs. By using new generation NGTs, designed for better aspiration but with the same, or even smaller, calibre as classical NGTs, it may become possible to more frequently avoid X-ray confirmation of NGTs insertion, without adding any additional burdens on the patients. The use of an electronic pH meter allows for more accurate measurements, which should translate to greater certainty in positioning as well as avoiding dubious results that may otherwise be an indication for X-ray. By checking correct positioning by X-ray we will further assess the sensitivity and specificity of pH testing for NGTs placement. Differentiation between the three possible latest-generation NGTs will not only look at aspiration potential, but also patient tolerability and adverse events, in order to avoid recommending NGTs that may turn out to be impractical in everyday clinical use. In particular, smaller calibre NGTs may allow for less patient discomfort, and subsequently greater compliance, ease of treatment and fewer nutrition-related adverse events. The main limitation of the study may be encountered with patients under antacid or PPI therapy, for which correct placement may give readings above pH 5.5, which are an indication for X-ray confirmation of correct placement. Unfortunately, no clinical trials exist that provide evidence on how to mitigate such factors, so we will gather data regarding possible confounding factors.

This study may not only improve the precision of NGTs placement, but could also have implications for the standardization of NGTs insertion protocols in medical institutions. It is worth noting that the knowledge gained from this study could affect and improve guidelines for NGTs placement in specific patient populations, particularly those receiving antacid or PPI therapy, where current practices are ambiguous. Furthermore, one key aspect of the study is its focus on measuring the time taken to start nutritional therapy after an NGTs placement request by a physician. This metric, quantified in minutes, is crucial in hospital nutritional care where timely delivery of nutrition can significantly impact patient recovery and quality of life. However, it has to be taken in consideration that the study's impact might be constrained by its focus on short-term outcomes, leaving an opportunity for future research to explore long-term implications and cost-effectiveness in diverse healthcare settings. Beyond the immediate clinical applications, this study's methodologies and findings could also have educational implications for clinical training and practice. By introducing new protocols for NGTs placement based on pH monitoring, there is an opportunity to revise and update training curricula for healthcare professionals, including nurses and physicians. This could lead to enhanced skill sets in NGTs management, educating a new generation of practitioners adept in utilizing advanced technologies for patient care.

## Sources of funding

The authors declare that there are no sources of funding

## CRediT authorship contribution statement

**Stefano Mancin:** Conceptualization, Data curation, Investigation, Methodology, Project administration, Supervision, Validation, Visualization, Writing – original draft, Writing – review & editing. **Pietro Stallone:** Conceptualization, Investigation, Writing – review & editing. **Valeria Siro:** Conceptualization, Methodology, Supervision, Validation, Writing – review & editing. **Manuela Pastore:** Conceptualization, Investigation, Methodology, Supervision, Writing – review & editing. **Daniela Cattani:** Visualization, Writing – review & editing. **Diego Lopane:** Validation, Writing – review & editing. **Alessandra Dacomi:** Validation, Writing – review & editing. **Francesco Carlo Tartaglia:** Writing – original draft, Writing – review & editing. **Alessandro Bellone:** Writing – original draft, Writing – review & editing. **Francesca Serazzi:** Writing – original draft, Writing – review & editing. **Georges Laffoucriere:** Writing – original draft, Writing – review & editing. **Chiara Coldani:** Visualization, Writing – review & editing. **Giuseppina Tomaiuolo:** Visualization, Writing – review & editing. **Beatrice Mazzoleni:** Methodology, Project administration, Supervision, Validation, Visualization, Writing – review & editing.

## Declaration of competing interest

The authors declare that they have no known competing financial interests or personal relationships that could have appeared to influence the work reported in this paper.

## References

[1] Norman K., Pichard C., Lochs H., Pirlich M. (2008 Feb). Prognostic impact of disease-related malnutrition. Clin. Nutr..

[2] Barker L.A., Gout B.S., Crowe T.C. (2011 Feb). Hospital malnutrition: prevalence, identification and impact on patients and the healthcare system. Int. J. Environ. Res. Publ. Health.

[3] Iliakis D., Kressig R.W. (2014 Jan). Malnutrition und Infekte [The relationship between malnutrition and immune]. Ther. Umsch..

[4] Trujillo E.B. (1993 Mar). Effects of nutritional status on wound healing. J. Vasc. Nurs..

[5] Landi F., Camprubi-Robles M., Bear D.E., Cederholm T., Malafarina V., Welch A.A., Cruz-Jentoft A.J. (2019 Oct). Muscle loss: the new malnutrition challenge in clinical practice. Clin. Nutr..

[6] Vong T., Yanek L.R., Wang L., Yu H., Fan C., Zhou E., Oh S.J., Szvarca D., Kim A., Potter J.J., Mullin G.E. (2022 Mar 21). Malnutrition increases hospital length of stay and mortality among adult inpatients with COVID-19. Nutrients.

[7] Curtis L.J., Bernier P., Jeejeebhoy K. (2017 Oct). Costs of hospital malnutrition. Clin. Nutr..

[8] Lew C.C.H., Wong G.J.Y., Cheung K.P. (2017 Dec 23). Association between malnutrition and 28-day mortality and intensive care length-of-stay in the critically ill: a prospective cohort study. Nutrients.

[9] Cederholm T., Bosaeus I., Barazzoni R. (2015 Jun). Diagnostic criteria for malnutrition - an ESPEN consensus statement. Clin. Nutr..

[10] Sharma Y., Thompson C.H., Kaambwa B. (2017 Oct 1). Investigation of the benefits of early malnutrition screening with telehealth follow up in elderly acute medical admissions. QJM.

[11] van den Berg G.H., Huisman-de Waal G.G.J., Vermeulen H. (2021 May). Effects of nursing nutrition interventions on outcomes in malnourished hospital inpatients and nursing home residents: a systematic review. Int. J. Nurs. Stud..

[12] Schneider S. (2022 Oct). Conduite thérapeutique à tenir devant une dénutrition [Therapeutic management of malnutrition]. Rev. Prat..

[13] Toulson Davisson Correia MI., Castro M. (2021 Sep). Nutrition therapy cost-effectiveness model indicating how nutrition may contribute to the efficiency and financial sustainability of the health systems. JPEN - J. Parenter. Enter. Nutr..

[14] Calder P.C. (2003). Immunonutrition: may have beneficial effects in surgical patients. Br. Med. J..

[15] Tan H.B., Danilla S., Murray A. (2014). Immunonutrition as an adjuvant therapy for burns. Cochrane Database Syst. Rev..

[16] Koontalay A., Suksatan W., Teranuch A. (2021 Sep 2). Early enteral nutrition met calories goals led by nurse on improve clinical outcome: a systematic scoping review. Iran. J. Nurs. Midwifery Res..

[17] Abela G. (2017 Oct). The potential benefits and harms of early feeding post-surgery: a literature review. Int. Wound J..

[18] Sun J.K., Mu X.W., Li W.Q. (2013 Feb 14). Effects of early enteral nutrition on immune function of severe acute pancreatitis patients. World J. Gastroenterol..

[19] Alexander J.W. (1990 Sep-Oct). Nutrition and translocation. JPEN - J. Parenter. Enter. Nutr..

[20] Marik P.E. (2011 Nov 10). Early or late parenteral nutrition in critically ill adults. N. Engl. J. Med..

[21] Singer P., Anbar R., Cohen J., Shapiro H., Shalita-Chesner M., Lev S., Grozovski E., Theilla M., Frishman S., Madar Z. (2011 Apr). The tight calorie control study (TICACOS): a prospective, randomized, controlled pilot study of nutritional support in critically ill patients. Intensive Care Med..

[22] Ling C., Hu X., Chen C. (2022 Sep). Comparison of nutritional effectiveness and complication rate between early nasojejunal and nasogastric tube feeding in patients with an intracerebral hemorrhage. J. Clin. Neurosci..

[23] EuroSurg Collaborative (2020 Dec). Timing of nasogastric tube insertion and the risk of postoperative pneumonia: an international, prospective cohort study. Colorectal Dis..

[24] Hamdaoui D., Ashworth J., Thompson J.D. (2022). A scoping review of clinical practices and adherence to UK national guidance related to the placement and position confirmation of adult nasogastric feeding tubes. Radiography.

[25] Barry J.M., Ling Relph W., Small M. (2020). A position paper on nasogastric tube safety. BAPEN. Sept.

[26] Ni M.Z., Huddy J.R., Priest O.H. (2017 Nov 4). Selecting pH cut-offs for the safe verification of nasogastric feeding tube placement: a decision analytical modelling approach. BMJ Open.

[27] Borsci S., Buckle P., Huddy J. (2017 Nov 30). Usability study of pH strips for nasogastric tube placement. PLoS One.

[28] Small M. (2017 Sep). Assessing the PH of aspirate from nasogastric tubes: are two heads better than one?. Clin. Nutr..

[29] Fan E.M.P., Tan S.B., Ang S.Y. (2017). Nasogastric tube placement confirmation: where we are and where we should be heading. Proc. Singapore Healthc..

[30] Milsom S.A., Sweeting J.A., Sheahan H. (2015 Sep). Naso-enteric tube placement: a review of methods to confirm tip location, global appli- cability and requirements. World J. Surg..

[31] Tho P.C., Mordiffi S., Ang E., Chen H. (2011 Mar). Implementation of the evidence review on best practice for confirming the correct placement of nasogastric tube in patients in an acute care hospital. Int. J. Evid. Base. Healthc..

[32] An E.M.P., Tan S.B., Ang S.Y. (2017). Nasogastric tube placement confirmation: where we are and where we should be heading. Proc. Singapore Healthc..

[33] Quek S., Junyang H., Ali A. (2018). Delays in nasogastric tube feeding in a tertiary stroke service. Int. J. Stroke.

[34] Al-Ali S., Mahon N., Walton G. (2016). Determining the frequency of repeat chest ra- diographs to establish NG tube position when no aspirate is obtainable - a retrospective study. Br. J. Oral Maxillofac. Surg..

[35] Spink T., Fennessy C., Bennell J. (2018). Frequency and outcome of radiological assessments for nasogstric tube placement. BAPEN Conf - Abstr Oral Commun..

[36] Hanna A.S., Grindle C.R., Patel A.A. (2012 Jan-Feb). Inadvertent insertion of nasogastric tube into the brain stem and spinal cord after endoscopic skull base surgery. Am. J. Otolaryngol..

[37] (2017 Aug). Nutrition Support for Adults: Oral Nutrition Support, Enteral Tube Feeding and Parenteral Nutrition.

[38] Chauhan D., Varma S., Dani M. (2021 Jan 21). Nasogastric tube feeding in older patients: a review of current practice and challenges faced. Curr Gerontol Geriatr Res.

[39] Hamdaoui D., Ashworth J., Thompson J.D. (2022). A scoping review of clinical practices and adherence to UK national guidance related to the placement and position confirmation of adult nasogastric feeding tubes. Radiography.

[40] Barry J.M., Ling Relph W., Small M. (Sept 2020). https://www.bapen.org.uk/pdfs/ngsig/a-position-paper-on-nasogastric-tube-safety-v2.pdf.

[41] An E.M.P., Tan S.B., Ang S.Y. (2017). Nasogastric tube placement confirmation: where we are and where we should be heading. Proc. Singapore Healthc..

[42] Hanna A.S., Grindle C.R., Patel A.A., Rosen M.R., Evans J.J. (2012 Jan-Feb). Inadvertent insertion of nasogastric tube into the brain stem and spinal cord after endoscopic skull base surgery. Am. J. Otolaryngol..

[43] Gilbertson H.R., Rogers E.J., Ukoumunne O.C. (2011). Determination of a practical pH cutoff level for reliable confirmation of nasogastric tube placement. JPEN - J. Parenter. Enter. Nutr..

[44] Rowat A.M., Graham C., Dennis M. (2018 Jun 9). Study to determine the likely accuracy of pH testing to confirm nasogastric tube placement. BMJ Open Gastroenterol.

